# Antibiotic Use in Hispanic Households, New York City

**DOI:** 10.3201/eid0909.020371

**Published:** 2003-09

**Authors:** Elaine Larson, Susan X. Lin, Cabilia Gomez-Duarte

**Affiliations:** * Columbia University School of Nursing, New York, New York, USA; † Mailman School of Public Health, Columbia University, New York, New York, USA

**Keywords:** antibiotics, antimicrobials, resistance, community health

## Abstract

Trained interviewers visited 631 inner city households to determine community prevalence and predictors of antibiotic use. Infectious disease symptoms were reported in 911 (33.2%) of 2,743 household members in the previous 30 days: medical attention was sought by 441 **(**48.4%) of 911 persons, and 354 (38.9%) of 911 took antibiotics for symptoms. Reported symptoms were respiratory (68.9%), gastrointestinal (15.3%), fever (12.8%), and skin infection (2.8%). Medical attention was sought significantly more often among those with chronic illness, those born in the United States, and those with fever, runny nose, or skin infections (all p<0.05). Antibiotics were taken significantly more often among those with poor health, those who spent more time at home, and those with fever and respiratory symptoms. Interventions to promote judicious use of antibiotics must include clinicians and the public, and for the Hispanic population such interventions must also be culturally relevant and provided in Spanish.

Evidence is overwhelming that antibiotic use is linked with increasing antimicrobial resistance in the community (*1*–*7*), even when biases and confounding are controlled for (*8*). Nevertheless, inappropriate prescribing and use of antimicrobial agents continue to be global problems. The reasons include public expectations and demand for medication and lack of understanding about the ineffectiveness of antibiotics against viral illness, the worldwide ease of access to antibiotics, the clinician’s wish to satisfy the patient, and pressure to weigh the interests of individual patients against the overall public health benefit (*9*–*11*). Data regarding antibiotic use have been gathered primarily from prescription information from primary care practices, despite the fact that antibiotics may be obtained in many communities by inter- and intra-household sharing or by other informal means. Thus, the use of antibiotics in the community may be underreported. In 1994, one community prevalence survey was conducted in Mexico in which homemakers were interviewed regarding the occurrence of diarrhea and use of antibiotics within the previous 2 weeks (*12*). However, we found no such studies which tracked antibiotic use among households in the United States. Therefore, we conducted a survey among 631 inner city households to determine self-reported prevalence and correlates of antibiotic use in Hispanic households.

## Methods

### Sample and Setting

The study was conducted in an upper Manhattan neighborhood with a >90% Hispanic population, who lived primarily in apartment complexes. The neighborhood was selected by convenience as part of a larger clinical trial to examine the role of home hygiene practices on the incidence of infectious disease symptoms because the neighborhood is densely populated with many households, including several generations, often with young children, living in close proximity. Participants were recruited by posters placed on local bulletin boards, brochures, and word-of-mouth in local pediatric clinics, community centers, and churches. To qualify for the study, households had to include at least three persons with at least one preschool child; study participants had to be able to converse in either Spanish or English and willing to allow the research staff to make a home visit. An initial group (n=398 households with 1,645 individual participants) was interviewed in a one-time home visit and prevalence survey from January 1999 to April 1999 (*13*), and a second group of households (n=238 with 1,177 participants) was surveyed between October 2000 and February 2001. Of those surveyed, 2,743 (97.2%) of 2,822 members of 631 households were Hispanic, and only those persons were included in this study. Fourteen households (2.2%) participated in both surveys.

### Procedures

The study was approved by the Institutional Review Board of Columbia Presbyterian Medical Center. Written informed consent was obtained from the primary caretaker in each household, usually the mother, by a member of the research team. This person served as the informant for all members of the household. Participants were extensively interviewed on a single occasion during a home visit of approximately 1 hour. We used a standardized interview form to obtain detailed demographic and health information was obtained about each household member, including whether one or more of seven symptoms of infection (vomiting, diarrhea, fever, runny nose, cough, sore throat, skin infections) had been present within the previous 30 days, and, if symptoms were present, whether medical treatment was sought, antibiotics were taken, or both, by any household member. Data regarding sore throat were obtained for persons >3 years of age only.

The questionnaire used in the home interview underwent extensive psychometric assessment and pilot testing (*14*). In the pilot work, we found high levels (>90%) of test-retest reliability and agreement between informant response and direct observation on a number of verifiable factors. On the basis of the pilot testing, a few items in the questionnaire were modified between the first and second survey. For example, in the first survey the specific country of origin was recorded, but since the majority of those born outside the United States. were from a single country, the Dominican Republic (1,391/1,520, 91.5%), the second survey asked only whether each participant was born outside or within the United States.

All interviews and home visits were conducted by one of four trained interviewers: three were physicians trained in the Dominican Republic and the fourth was a community health interviewer from Guatemala. All were native Spanish speakers. Interviewers underwent an extensive orientation process and were monitored throughout the entire study by the project director, who accompanied the interviewers on a random sample of 10% of home visits. No information about socioeconomic status, household income, or insurance status was requested, but the population was quite homogeneous because the study was conducted in a single neighborhood of predominantly working poor and middle-class immigrants. Participants were given the option of having the interview in Spanish or English; all chose Spanish.

As part of the larger study, we attempted to verify the validity and reliability of the self-reported symptoms in the following manner. For the first 100 reports of any symptom during the second round of interviews, one of the physician interviewers made an additional home visit for the purpose of directly observing the symptom (e.g., if fever was reported, the temperature was taken, if vomiting or diarrhea was reported, the output was examined; if sore throat was reported, the throat was examined). In 93% of cases, directly verifying the presence of the reported symptoms was possible. Further, in 3% of visits a symptom was identified by the interviewer that had not been reported. Hence, the sensitivity and specificity of the symptom self-reports were 93% and 97%, respectively. To verify the accuracy of reporting of antibiotic usage, participants were asked to give the name of the antibiotic and, when available, the labeled bottle or envelope containing the antibiotic was examined by the interviewer. Interviewers only recorded that an antibiotic was taken when the informant was able to give the specific name or when the package label was examined and confirmed to be an antibiotic.

### Data Analysis

Analyses were conducted only on Hispanic participants at the level of the individual household member. Chi square and Student t tests were used to compare demographic and illness variables between the two survey periods. Then, for all participants combined, logistic regression models were fit to identify predictors of seeking medical attention and taking an antibiotic among those with an infectious disease symptom. Independent variables used in the models included age, sex, health status (excellent/good vs. fair/poor), presence of chronic illness, household (since symptoms and practices were likely to be interdependent among members of the same household), type of symptom, time spent outside the home, and whether born outside the United States. A two-sided p value of <0.05 was considered statistically significant.

## Results

Characteristics of the participants (1,586 persons in survey 1; 1,157 in survey 2) are summarized on Table 1. Approximately one third of household members were 5 years of age or younger (27.4%), 56.3% were women, 55.5% born outside the United States, and most reported excellent or good health (80.6%) with no chronic illnesses (85.9%). About one third spent >40 hours/week outside the household (35.3%), and another 35.1% spent >20 hours/week outside the household. No significant differences were found between survey 1 and survey 2 with regard to participants’ sex (p=0.09), mean ages (20.6 years in survey 1 and 20.2 years in survey 2; p=0.51), or proportion born outside the United States (p=0.16). Significantly more persons in survey 1 had fair/poor health (p<0.001), chronic disease (p=0.006), and spent >40 hours/week outside the home (p<0.001) than in survey 2.

**Table 1:** Characteristics of 2,743 Hispanic household members

Characteristic	Survey 1 Jan. 4, 1999 (%)	Survey 2 Oct. 2000–Feb. 2001 (%)	Total
Sex Female Male Unknown	914 (57.6) 672 (42.4)	629 (54.4) 528 (45.6)	1543 (56.3) 1200 (43.7) 0
Age (y) 0–5 6–10 11–19 20–35 36–45 46–60 >60 Unknown	405 (25.6) 228 (14.4) 208 (13.1) 414 (26.1) 196 (12.4) 111 (7.0) 23 (1.7) 1 (0.06)	347 (30.0) 130 (11.2) 119 (10.3) 335 (29.0) 137 (11.8) 69 (6.0) 20 (1.7) 0	752 (27.4) 358 (13.1) 327 (11.9) 749 (17.3) 333 (12.1) 180 (6.6) 43 (1.6) 1 (0.04)
Country of birth United States Outside United States Unknown	687(43.3) 899 (56.7)	530 (46.0) 621 (54.0)	1217 (44.5) 1520 (55.5) 6 ( 0.2)
Health status^a^ Excellent or good Fair or poor Unknown	1259 (79.4) 326 (20.6)	953 (83.2) 193 (16.8)	2212 (80.6) 519 (18.9) 12 ( 0.4)
Chronic illness^a^ Yes No Unknown	248 (15.6%) 1338 (84.4%)	133 (11.9%) 984 (88.8%)	381 (14.1) 2322 (85.9) 0
Time spent outside the home/week ^a^ 40+ hours/week 20–40 hours/week <20 hours/week Unknown	469 (29.6) 508 (32.1) 607 (38.3)	497 (43.1) 302 (26.2) 355 (30.8)	955 (35.3) 810 (29.5) 962 (35.1) 16 (0.6)
Prevalence of infectious disease symptoms in previous 30 days Cough^a^ Runny nose^a^ Sore throat (only in age ≥3 y)^a^ Fever^a^ Vomiting^a^ Diarrhea^a^ Skin infection^a^ Total symptoms	371 (24.6) 369 (24.5) 273 (18.1) 205 (13.6) 134 (8.9) 102 (6.8) 54 (3.6) 1,508	150 (31.6) 142 (29.9) 61 (12.8) 49 (10.3) 39 (8.2) 32 (6.7) 2 (0.4) 475	521 (26.3) 511 (25.8) 334 (16.8) 254 (12.8) 173 ( 8.7) 134 (6.6) 56 ( 2.8) 1,983
Persons with at least one symptom^a^	662/1,586 (41.7)	249/1,157 (21.5)	911/2,743 (33.2)
If symptoms present Sought medical attention for symptoms^a^	353 (53.3)	88 (35.3)	441 (48.4)
Received some treatment^a^	345 (52.1)	41 (16.5)	387 (42.5)
Took antibiotics^a^	257 (38.8)	97 (49.5)	354 (38.9)

^a^Significantly different between two survey periods chi square, all p<0.01.

### Prevalence of Symptoms and Antibiotic Use

Among the 2,743 household members, 911 (33.2%) reported 1,983 symptoms in the previous 30 days, the most common being respiratory (runny nose, cough, and sore throat, 68.9%), followed by gastrointestinal (vomiting, diarrhea, or both, 15.3%), fever (12.8%), and skin infection (2.8%). Approximately half of persons with infectious disease symptoms (441/911, 48.4%) sought medical attention for the symptoms, 42.5% received some type of treatment, and 38.9% took an antibiotic. Respondents in survey 1 reported significantly more symptoms than those in survey 2 (p<0.001) (Table 1).

### Predictors of Seeking Medical Attention and Taking Antibiotics

Among those with one or more infectious disease symptom, we found no significant differences in either seeking medical attention or taking antibiotics by age (p=0.07 and 0.56, respectively), gender (p=0.25 and 0.44, respectively), or by presence of gastrointestinal symptoms (vomiting or diarrhea, all p>0.20). As expected, household number was significantly associated with both seeking medical attention and taking an antibiotic. Those born in the United States were significantly more likely to seek medical attention (odds ratio [OR] 2.1; 95% confidence limits [CL] 1.53 to 2.80; p<0.001) but not to take an antibiotic. Chronic illness or poor health status was a significant predictor of seeking medical attention and of taking an antibiotic. Finally, those who spent less time at home were significantly less likely to report symptoms of infection (28.6% of those outside the home >40 hours/week compared with 35.3% outside the home <20 hours/week had symptoms; p=0.006) and less likely to receive antibiotics (OR 0.63; 95% CL 0.43 to 0.92; p=0.02).

Several symptom complexes were predictive of seeking medical attention, taking an antibiotic, or both. Those with skin infections were significantly more likely to seek medical attention (OR 2.70; 95% CL 1.53 to 4.78; p=0.001) but not to take antibiotics. Those with runny nose and fever were significantly more likely to seek medical attention and take antibiotics; those with cough and sore throat were also significantly more likely to take antibiotics. The regression models with the best fit for seeking medical attention and taking an antibiotic are summarized in Table 2.

**Table 2:** Significant predictors of seeking medical attention and taking antibiotics among those with symptoms^a^

Predictor	Odds ratio (95% confidence limits)	p value^b^
**Seeking medical attention**		
Household	0.995 (0.994 to 0.996)	<0.001
Chronic illness	2.01 (1.43 to 2.85)	<0.001
Born outside United States	2.07 (1.53 to 2.80)	<0.001
Skin infection	3.06 (1.65 to 5.69)	<0.001
Fever	2.55 (1.82 to 3.57)	<0.001
Runny nose	1.44 (1.06 to 1.96)	0.02
**Taking an antibiotic**		
Household	0.997 (0.996 to 0.998)	<0.001
Excellent/good health	0.44 (0.28 to 0.70)	<0.001
Outside household ≥40 h/week	0.59 (0.41 to 0.86)	0.007
Fever	2.39 (1.72 to 3.32)	<0.001
Runny nose	1.38 (1.01 to 1.88)	0.04
Sore throat	2.44 (1.80 to 3.31)	<0.001
Cough	1.42 (1.05 to 1.94)	0.03

^a^n=911.

^b^Logistic regression.

## Discussion

### Prevalence of Antibiotic Use

The impact of illness with infectious disease symptoms in this community seemed high with about one third of household members reporting at least one symptom within the previous 30 days. However, determining how this compares with the prevalence of such symptoms in other generally healthy community populations is not possible since surveillance systems for infections are either hospital-based or physician-based (*15*), and most infections in the community never come to the attention of a healthcare provider. Respondents in survey 1 reported more symptoms than respondents in survey 2, perhaps because significantly more persons reported chronic diseases and poor health during survey 1. However, this difference may reflect some seasonal variation; survey 1 was carried out primarily through the winter and early spring; survey 2 was conducted primarily during the fall and early winter.

Of those who reported symptoms, about half sought medical attention and over one third took an antibiotic. In fact, antibiotic usage was higher in this New York City neighborhood than in a periurban community in Mexico City over a decade ago, in which 5% of 8,279 persons reported using an antibiotic during the previous 2 weeks (*16*). The prevalence of antibiotic use in our study likely represents overuse, since most symptoms reported were indicative of acute respiratory viral illness (runny nose, cough, sore throat), and antibiotics are inappropriate for such infections (*17*,*18*). This finding is disappointing in light of the increased attention among primary care providers and in the public media to the problem of antibiotic resistance and the need for prudent use of antimicrobial agents (*4*,*7*,*19*). Although antibiotic resistance among agents that cause community-acquired respiratory tract infections seems to be increasing in the Americas, and particularly in Mexico (*20*), a recent national survey of generalist physicians found that the issue of contributing to antibiotic resistance was the lowest determinant of their choice regarding antibiotic prescribing for patients with community-acquired pneumonia (*9*).

As with all self-reported data, this survey contains the potential for recall bias and underreporting or overreporting. Although the answer would be useful to know, we chose not to ask respondents whether the antibiotics they took were obtained by prescription to discourage underreporting. One of the weaknesses of this study is that some participants were reluctant or unable to show the antibiotic container and label to the interviewer, perhaps because antibiotics were not always obtained by written prescription or were borrowed from others. In the study neighborhood, we verified that antibiotics were available over the counter in local *bodegas* (small stores). Households also informally reported sharing antibiotics between family members or among friends. Although the interviewers did not record the specific name of antibiotic after verifying that the medication was, in fact, an antibiotic, clearly, the antibiotics taken were frequently obtained without prescription. Another limitation of the study was that we were unable to examine differences between subgroups of the Hispanic population, since most respondents in this study were from a single country.

Our findings have several important implications. First, estimates of antibiotic usage that are based on prescription data are likely to result in considerable underreporting of antibiotic usage in the community. Second, this generally unaccounted-for usage may be a major contributor to the ongoing problem of antibiotic resistance in the community. Few studies have investigated prevalence of use of unprescribed antibiotics. Among a Mexican immigrant population in Los Angeles, 28% reported obtaining medication, frequently antibiotics, in Mexico (*21*). Antibiotic use without prescription is certainly not limited, however, to immigrant or Hispanic populations. Among 1,363 primarily Anglo-American and college-educated adults seen in a New Jersey emergency department, 22% reported that their physicians routinely prescribed antibiotics for cold symptoms, and 17% reported taking antibiotics left over from previous prescriptions, primarily for respiratory symptoms (*22*). Further, parents who lived in a suburban site were more likely than urban parents to have misused antibiotics, but urban parents were more likely to have been discharged from one health facility and gone to another office or emergency department to obtain antibiotics for their children (*23*).

To be effective within populations such as the one in this study, media and public health efforts to educate the public on the prudent use of antibiotics must be circulated widely in Spanish and other languages appropriate for recent immigrants, particularly those from countries where antibiotics are widely available without prescription. Further, the messages conveyed must be culturally meaningful for such targeted subgroups. The households in this study all spoke Spanish as their primary language. Language barriers, particularly among Hispanics, have been found to be associated with higher costs of care in the emergency department (*24*) and Spanish-speaking Hispanics in the United States have a larger gap in immunization rates than do English-speaking Hispanics (*25*,*26*).

### Correlates of Antibiotic Use

Persons with poor health or a known chronic condition were more likely to receive medical attention and antibiotics. The fact that more U.S.-born persons sought medical attention may relate to insurance coverage or other access issues. Although we did not ask respondents about their insurance or immigration status in this study, in a previous survey of this community, Hispanics reported particularly low participation in insurance plans; 47% who had lived in the United States <5 years were uninsured, and the major reported reason for not seeking care was financial concern (*27*). In one study, conducted in Colorado and Utah, neither Hispanic ethnicity nor payment source was associated with antibiotic prescription rates (*28*), but other studies have shown lower insurance rates among children of immigrant parents than among those with U.S.-born parents (*29*). Several surveys have shown that Hispanics have fewer encounters with the healthcare system and are less likely to receive preventive care than Anglo-Americans (*30*,*31*).

On the one hand, this study showed a possible improvement in specific antibiotic-taking practices. A study conducted almost 10 years ago reported that 60% of patients seeing a physician for the common cold filled a prescription for an antibiotic (*10*). In our study, a younger age of patients, when other factors were controlled for in the regression analyses, was not associated with increased likelihood of taking antibiotics. These findings are consistent with a recent report from the National Ambulatory Medical Care Survey that demonstrated a reduction in population-based and visit-based antimicrobial prescriptions for persons younger than 15 years of age 40% and 29%, respectively (*32*).

On the other hand, since a large proportion of persons in this study took an antibiotic for symptoms that were likely to be viral in origin, antibiotic overuse seems likely. A number of factors may contribute to a continued overuse of antibiotics. First, even though antibiotic prescribing for children with upper respiratory infection improved during the decade of the 1990s (*32*), 20% to 50% of antibiotic prescriptions in the community are considered unnecessary (*10*,*33*,*34*). Antibiotics are still prescribed by primary care physicians for most patients seen with respiratory illnesses such as a sore throat, bronchiolitis, or “upper respiratory tract illness” (*35*,*36*).

Inappropriate use of antibiotics in this Hispanic population may also relate to practices in their country of origin. McKee et al. reported that persons from countries where antibiotics are readily available over the counter are more likely to use antibiotics not prescribed by clinicians; about one fourth of 192 persons surveyed in an ethnically diverse urban community in New York City obtained antibiotics from sources other than prescription (*11*). Further, a summary from the SENTRY Antimicrobial Surveillance Program reported that gram-negative isolates from Latin America were uniformly more resistant to all classes of antimicrobial agents than isolates from North America (*37*). Not surprisingly, persons in this study who spent less time at home were less likely to be ill and to take antibiotics. Additionally, a recent study has confirmed that most cases of influenza in households in which one member was infected resulted from secondary transmission within the household rather than acquisition from community sources (*38*). Thus, those who spend more time at home are at higher risk for secondary transmission.

Interventions directed at providers and patients to promote more judicious use of antibiotics have met with variable success. Some trials have included both clinicians and patients or the patient’s parents (*39*,*40*), and others have focused primarily on clinicians (*41*,*42*). After considering these studies, we note that interventions involving only the patients or the public are not successful; providers must also be included. Clinicians are resistant to changing their behavior (*43*), and antibiotic-prescribing patterns are a particular challenge because of pressure from patients or their family members for antibiotics. In fact, one of the most successful interventions reported to date was a comprehensive, communitywide educational endeavor directed to all three audiences—providers, parents, and patients. In that study, an 11% decrease in prescription rates (which was greatest among prescriptions for children 1 to 5 years of age) could be attributed to an intervention (*44*).

In summary, about one third of members of this Hispanic community reported infectious disease symptoms over a 30-day period, and antibiotics were taken by more than one third when a symptom was present. When controlled for other factors in the logistic regression analyses, age was not a predictor of seeking medical attention or of taking antibiotics. Persons with fever, runny nose, or skin infection; persons born in the United States; and those with chronic disease were significantly more likely to seek medical attention. Those with respiratory symptoms and poor health and those who spent more time at home were significantly more likely to take antibiotics. Continued interventions to promote judicious use of antibiotics must include both clinicians and the public; for the Hispanic population, such interventions must also be culturally relevant and provided in Spanish.
